# Bioimaging of C2C12 Muscle Myoblasts Using Fluorescent Carbon Quantum Dots Synthesized from Bread

**DOI:** 10.3390/nano10081575

**Published:** 2020-08-11

**Authors:** Karthiga K. Anpalagan, Jimsheena V. Karakkat, Adam Truskewycz, Ahmed Al Saedi, Paul Joseph, Vasso Apostolopoulos, Kulmira Nurgali, Ivan Cole, Zibo Cai, Daniel T. H. Lai

**Affiliations:** 1 Institute of Health and Sport (IHES), Victoria University, Melbourne, VIC 3011, Australia; Jimsheena.ValiyakathKarakkat@vu.edu.au (J.V.K.); vasso.apostolopoulos@vu.edu.au (V.A.); Kulmira.Nurgali@vu.edu.au (K.N.); zibo.cai@live.vu.edu.au (Z.C.); daniel.lai@vu.edu.au (D.T.H.L.); 2 Advanced Manufacturing and Fabrication, School of Engineering, Royal Melbourne Institute of Technology (RMIT), Melbourne, VIC 3000, Australia; adam.truskewycz@rmit.edu.au (A.T.); ivan.cole@rmit.edu.au (I.C.); 3 Australian Institute for Musculoskeletal Science (AIMSS), The University of Melbourne and Western Health, St. Albans, VIC 3000, Australia; ahmed.mohan@unimelb.edu.au; 4 Institute of Sustainable Industries and Liveable Cities, Victoria University, Melbourne, VIC 3011, Australia; paul.joseph@vu.edu.au

**Keywords:** carbon quantum dots, green synthesis, bioimaging, C2C12 muscle myoblast

## Abstract

Biocompatible carbon quantum dots (CQDs) have recently attracted increased interest in biomedical imaging owing to their advantageous photoluminescence properties. Numerous precursors of fluorescent CQDs and various fabrication procedures are also reported in the literature. However; the use of concentrated mineral acids and other corrosive chemicals during the fabrication process curtails their biocompatibility and severely limits the utilization of the products in cell bio-imaging. In this study; a facile; fast; and cost-effective synthetic route is employed to fabricate CQDs from a natural organic resource; namely bread; where the use of any toxic chemicals is eliminated. Thus; the novel chemical-free technique facilitated the production of luminescent CQDs that were endowed with low cytotoxicity and; therefore; suitable candidates for bioimaging sensors. The above mentioned amorphous CQDs also exhibited fluorescence over 360–420 nm excitation wavelengths; and with a broad emission range of 360–600 nm. We have also shown that the CQDs were well internalized by muscle myoblasts (C2C12) and differentiated myotubes; the cell lines which have not been reported before.

## 1. Introduction

In recent years, quantum dots (nanoparticles having typical diameters of ≤10 nm), have gained an increasing interest in a variety of photonic applications, such as chemical sensors, biosensors, light-emitting diode (LED), and electrocatalysis [1,2,3,4,5,6]. Carbon quantum dots (CQDs) are a relatively new family of nanoparticles, with carbon cores and associated ligands that often exhibit unique optical properties, and thus have emerged as the most promising materials for biomedical applications, especially, given their enhanced bio-compatibility [7]. CQDs generally show strong absorption in the ultra-violet region and exhibit excitation-dependent emission in the visible spectrum, an interesting property for the purpose of bioimaging [8,9,10,11]. The synthetic techniques for the fabrication of CQDs generally fall into two categories, namely top-down and bottom-up approaches. The top-down method essentially breaks apart a bulk material of interest into particles of nanometer dimensions under chemically harsh conditions, such as treatments with concentrated acids and at relatively high temperatures [12,13,14]. On the other hand, the bottom-up method fabricates CQDs from molecular precursors that include citric acid, glucose, sucrose, phloroglucinol, and formamide [15,16,17,18,19,20,21]. However, most of the currently practiced techniques are not environmentally friendly (i.e., “green”) because of the usage of harsh acids, base, or solvents with synthetic chemical precursors, and thus pose risks when used as fluorescent markers in cell bio-imaging. Environmentally sustainable routes have been reported recently for carbon dot fabrication from spent coffee grounds [22] with relatively good quantum efficiencies. Therefore, it is prudent to seek and explore more novel and environmentally benign routes to fabricate CQDs with minimal cytotoxicity attribute.

An environmentally friendly fabrication process could begin by breaking down polymeric organic material, or a natural product, into CQDs. Previously, carbon nanoparticles were fabricated from bread [23,24] and also from starch-rich resources, such as rice, potato, cassava, and yam [25]. Generally, mineral acids and/or fine chemicals were utilized in all of these fabrication methods; for example, a sample obtained from the charred bread was treated with nitric acid for oxidation, or in the case of other samples, treatments with toxic lower aliphatic alcohols, like methanol, was necessary. Concentrated phosphoric acid (14.6 M) and sulfuric acid (2 M) were also often used as passivating agents during the CQD synthesis from starch [25].

The synthesized CQDs, through different strategies, have been extensively explored for application as fluorophores in bioimaging and bio-diagnostic fields. Compared to the current fluorescent dyes, which are expensive and toxic, CQDs have the unique features of bio-compatibility, non-toxicity, tunable photoluminescence, excellent photostability, chemical stability, physicochemical stability, water-solubility, non-blinking characteristics, and thus are potentially superior candidates for cell and tissue imaging [26,27,28]. The surface chemistry and ligands of these carbon nanoparticles enhance bioconjugation with antibodies, proteins, or small molecules, that allow CQDs to be targeted biomarkers to detect specific proteins e.g., inflammation factors [29]. Several reports have already successfully demonstrated the applicability of CQDs synthesized through different techniques to image HeLa cells, A549, L02 cells, and macrophage cells [30,31]. 

Here, we report for the first time on the synthesis of CQDs from bread samples, primarily through a thermal carbonization technique, where no chemical compounds were used. The CQDs thus obtained through a greener, facile, and cost-effective method were found to be fluorescent over a wide range of excitation wavelengths without even requiring further chemical doping. To investigate the efficacy of the prepared CQDs, C2C12, the muscle myoblast cell line, which has the capacity to form myotubes in vitro [32] were selected. To the best of our knowledge, the present investigation is the first study of its kind, where a 2-D cell culture model system for bio-imaging by CQDs was employed. The internalization of these CQDs by C2C12 myoblast, as well as mature myotubes, was evident by using a microscopic fluorescence probe.

## 2. Materials and Methods

### 2.1. Fabrication of CQD Samples

In the present study, white toast bread (sourced from a local supermarket) was used for CQD fabrication by using two different methods. Typically, 100 g of bread contains 49 g of carbohydrate, 3 g of sugar, 8 g of protein, 1.9 g of fat, 2.7 g of dietary fiber, and 400 mg of sodium. In the first method, a domestic bread toaster (TARSST19B, Target Corporation, Williams Landing, Australia, operating at 220–240 V, 50 Hz, 780–830 W) was used to toast two slices of bread (56.57 g ± 0.1 mg) over different time intervals starting from 120 s to 300 s. Here, various temperature settings from 100–160 °C were also employed with a view to investigating the effect of the carbonization process on the nature of the resulting precursors to the CQDs. The appropriate time duration and temperature were identified as 255 s and 160 °C, respectively, after the samples were characterized, and optimum photoluminescence observed. The carbon residues from the charred part of the bread slice were carefully scrubbed out, and ground using mortar and pestle until a fine powder of 3.64 g (±0.1 mg) was obtained. About 0.5 g (±0.1 mg) of fine carbon powder was dispersed in 50 mL of Milli-Q water by sonication (Soniclean, LABOUIP Technologies, Bayswater, Australia) for 5 min, and the mixture was then centrifuged (MSE centrifuge, Thomas Scientific, New Jersey, USA) for 5 min at 3000 rpm. The resulting supernatant was filtered using a number 1 (90 mm) filter paper (Whatman, GE Healthcare UK limited, Amersham, UK). A sterile syringe filter unit (Minisart, Sartorius, Gottingen, Germany) of 0.2 µm was used to purify the sample solutions and prevent bacterial growth. This sample was labelled CQD-A.

In the second method, the two bread slices of 56.52 g (±0.1 mg) were cut into smaller pieces and transferred into a ceramic crucible preheated in a muffle furnace (SurTec, SUNVIC, Hamilton, UK) at 60 °C. The bread pieces were carbonized for 30 min and removed from the furnace at 220 °C. The bread pieces were then carbonized at assorted temperatures, starting from 180 °C to 300 °C for several time intervals starting from 20 min to 90 min, to enhance the technique and optimize the procedure. The optical characterization and the photoemission result also helped to identify the suitable procedure, which was then followed throughout the investigation. The dark brown colored product that was obtained from the muffle furnace was ground using mortar and pestle until a fine powder of 8.21 g (±0.1 mg) was obtained. Another sample was prepared from this fine carbon powder using the same purification procedure used for CQD-A. This sample was labelled CQD-B.

### 2.2. Chemical and Optical Characterization of CQD Samples

#### 2.2.1. Transmission Electron Microscopy (TEM)

The lyophilized samples were prepared by resuspending them in ultrapure water (18 MΩ) and filtering through a 0.2 µm filter. The filtrate was dropped onto a holey-carbon grid and allowed to dry. Morphological characteristics of the particles were observed using a JEOL 1010 TEM (JEOL Ltd., Tokyo, Japan) operated at an accelerating voltage of 100 kV.

#### 2.2.2. Fourier Transform Infrared Spectroscopy (FTIR)

FTIR spectra of the lyophilized samples were determined by Fourier transform infrared spectroscopy (Perkin Elmer, Waltham, MA, USA). An average of 16 scans with a resolution of 4 cm^−1^ was performed within the range of 4000–400 cm^−1^.

#### 2.2.3. Nuclear Magnetic Resonance Spectroscopy (NMR)

With a view to obtaining the chemical natures of the CQD materials, we employed a Bruker 600 MHz NMR instrument (Billerica, MA, USA) and the ^1^H and ^13^C spectra were run in deuterated water (D_2_O) at ambient probe conditions. In the case of the proton spectrum, an in-built spectral editing technique was used to “suppress” the undesirable and otherwise prominent residual proton signal from water. The collected spectra were then processed by proprietary software from Bruker (TopSpin, version 4.0.8; Software for Processing the Acquired NMR Data; Bruker plc, Melbourne, Australia, 2016).

#### 2.2.4. X-Ray Diffraction (XRD)

The extent of crystallinity of the lyophilized samples was determined by a diffractometer, XRD (Bruker AXS D8 DISCOVER, Billerica, MA, USA), equipped with a Cu Kα radiation source (λ = 1.5418 Å) operating at 40 kV and 35 mA. Spectral data were attained in the μ-2θ locked-couple mode over a 2θ interval of 5–90°.

#### 2.2.5. Zeta Potential

Particle surface charges were measured in ultrapure water (18 MΩ) using a Malvern 2000 Zetasizer, Malvern, UK following appropriate dilution and sonication within a DTS 1060C cuvette (Malvern, UK).

#### 2.2.6. Fluorescence Measurements

Fluorescence measurements were carried out on a CLARIOstar^®^ (BMG LABTECH, Ortanberg, Germany) 96-welled plate reader with a Costar^®^ 96-Well Black Polystyrene Plate (Ortanberg, Germany). Maximum excitation and emissions were determined by scanning aqueous samples (150 μL) from 320 to 520 nm in 20 nm increments. Emissions spectra were recorded between 20 nm above the excitation value to 700 nm.

### 2.3. Cell Culture

The synthesized CQDs were tested on C2C12 mouse muscle myoblasts (ATCC^®^ CRL-1772™ via Sigma, Australia) to evaluate their potential in bio-imaging of cells. C2C12 cells were routinely maintained in DMEM media containing 10% FBS (fetal bovine serum) and 1x Antibiotic-Antimycotic (Gibco^®^ Catalog number: 15240062) and kept at 37 °C in a humidified, 5% CO_2_ atmosphere. The cells were seeded on to coverslips pretreated with poly-L-lysine and after 24 h, CQDs (185 µg/mL) were added to the media at 1:2 (CQDs:media (volume/volume)) ratio and incubated for the specified amount of time along with the control cells. The control cells were treated with an equal volume of sterile water. Cytotoxicity of CQDs was tested by seeding equal number of cells in 6-well plates and treating them with CQDs, or water. After 24 h of incubation, the cells were lifted by treating them with 1× TrypLE™ Select enzyme for 5–10 min and diluted with the complete media. The cells (0.1 mL) were treated with an equal volume of trypan blue solution (0.4%) and viable cells were counted using hemocytometer and calculated. Cells from four individual wells were counted in duplicates for each sample set for representation.

Differentiation was initiated by washing 90–95% confluent cultures with phosphate buffered saline (PBS) and incubation in differentiation media (DM: DMEM with 2% horse serum and 1% PS), for 3–5 days with media change every 24 h. The differentiated myotubes were treated with CQDs as described above for 24 h.

For imaging, all coverslips with cells were fixed with 4% formalin for 15 min, washed with PBS, and mounted on slides with or without DAPI. The cells were visualized with a fluorescent microscope (Nikon-Tish-A1R-MP, Melville, USA) at 20× setting. All images were converted to the tagged information file format and processed with the Adobe Photoshop program (Photoshop CC 2015, Adobe, San Jose, CA, USA).

## 3. Results and Discussions

### 3.1. Characterization of CQD Samples

**Figure 1:**
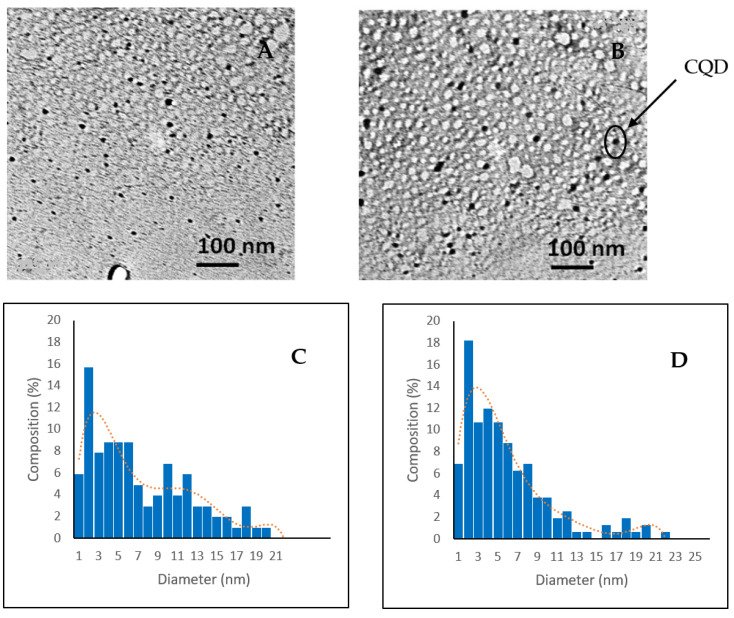
TEM images and size distribution histograms. (**A**,**C**) TEM image and size distribution histogram of carbon quantum dots CQD-A; (**B**,**D**) TEM image and size distribution histogram of CQD-B.

Transmission electron microscopy (TEM) was performed to determine the morphological characterization. Figure 1 shows the TEM images and the size distribution histogram of the lyophilized samples for both CQD-A and CQD-B. The presence of spherical quantum dots is clearly visible in both samples. Most of the nanoparticles are less than 10 nm in diameter. The contrast enhanced images of TEM are available in Supplementary Materials (Figure S1).

Considering the fact that most previous reports relied on environmentally hazardous chemicals to successfully fabricate CQDs, here we follow the green route of synthesis and hence potentially safe for most downstream applications like bio-imaging. It is important to note that this particle size was obtained with a green route of synthesis without any environmentally hazardous chemicals.

**Figure 2:**
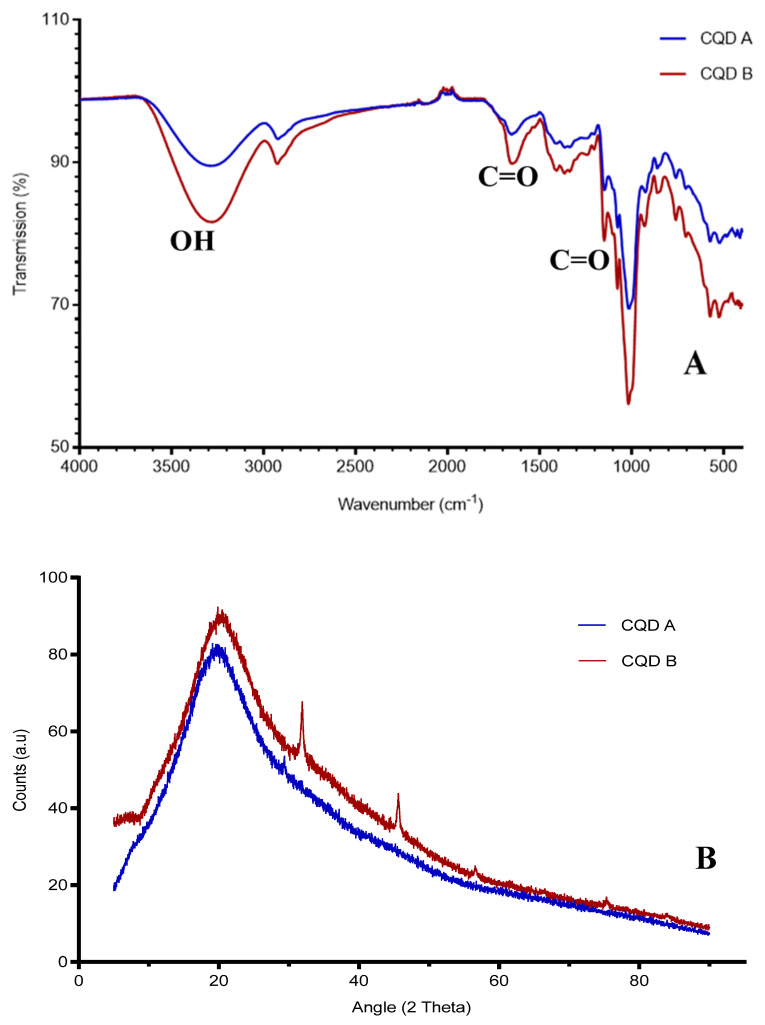
(**A**) FTIR spectrum of CQD-A and CQD-B; (**B**) XRD pattern of CQD-A and CQD-B.

FTIR analysis (Figure 2) shows that the overall vibrational spectral pattern very closely resembled that of the parent carbohydrate (i.e., starch molecule). The broad peak at between 3200 and 3500 cm^−1^ can be attributed to the hydroxyl group (-OH), either from the unburned starch matrix or from terminal hydroxyls attached to the graphitic carbons. The presence of the carboxyl (-COOH) group is clearly visible through C=O stretching (around 1653 cm^−1^, 1700 cm^−1^, and 1200 cm^−1^).

The structural and morphological features of the synthesized samples were primarily elucidated through X-ray diffraction studies (Figure 2). The XRD spectra of the two samples showed negligible well-defined peaks indicating that the conditions generated during pyrolysis did not favor the production of crystalline carbon dots. Even though both samples confirmed their identical nature, CQD-A is more amorphous (80.2%) than the CQD-B (74%). The broad diffraction peaks were seen at 21 degree for both samples. This was comparable with previous reports [33] and was in alignment with JCPDS 41-1487 (graphite).

**Figure 3:**
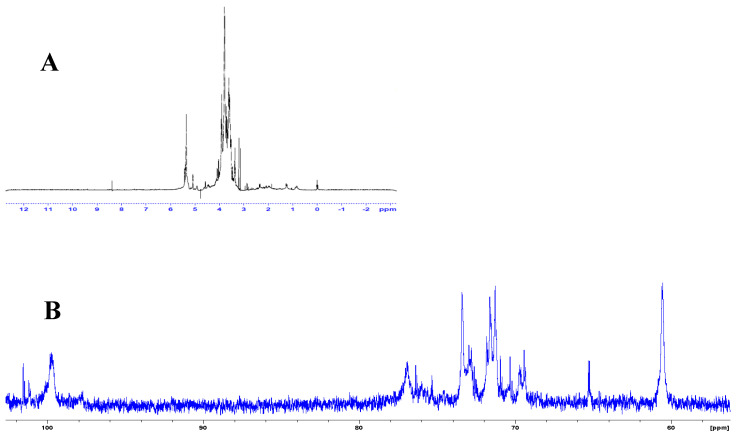
(**A**) ^1^H spectrum of a colloidal solution of the CQD in D_2_O; (**B**) the corresponding ^13^C spectrum.

In the NMR spectra (Figure 3A,B), there is evidence regarding the chemical environments of protons, as well as the chemical nature/hybridization states of the carbon atoms to which the protons are attached [34,35]. These broadly agree with the complementary information obtained through the FT-IR spectra. The signals in the ^1^H spectra (Figure 3A) can be assigned as follows: 1–3 ppm (H attached to sp^3^ carbons); 3–6 ppm (for the protons attached to oxygenated, such as hydroxyl and ether, and carbonyl groups; 8–10 ppm (aldehydic protons). In the corresponding ^13^C NMR spectrum (Figure 3B), at least three corresponding signals/regions can be unambiguously identified: 20–80 ppm (sp^3^ carbons and carbons bonded hydroxyl groups); 80–100 ppm (for carbons attached to ether functions); 100–120 ppm (aromatic, or sp^2^ carbons). 

Zeta potential, which is indicative of particle surface charge, is widely used to characterize nanometer-sized particles in the dispersion [36] and analyze particle colloidal stability. The particle surface charge of samples CQD-A and CQD-B is −10.42 mV and −7.24 mV, respectively. The stability of the colloidal dispersions generally increases with the magnitude of the zeta potential [37]. The zeta potential of the cells should be greatly negative (membrane potential of −50.5 ± 0.8 mV) and the particles show only very weak stability. 

**Figure 4:**
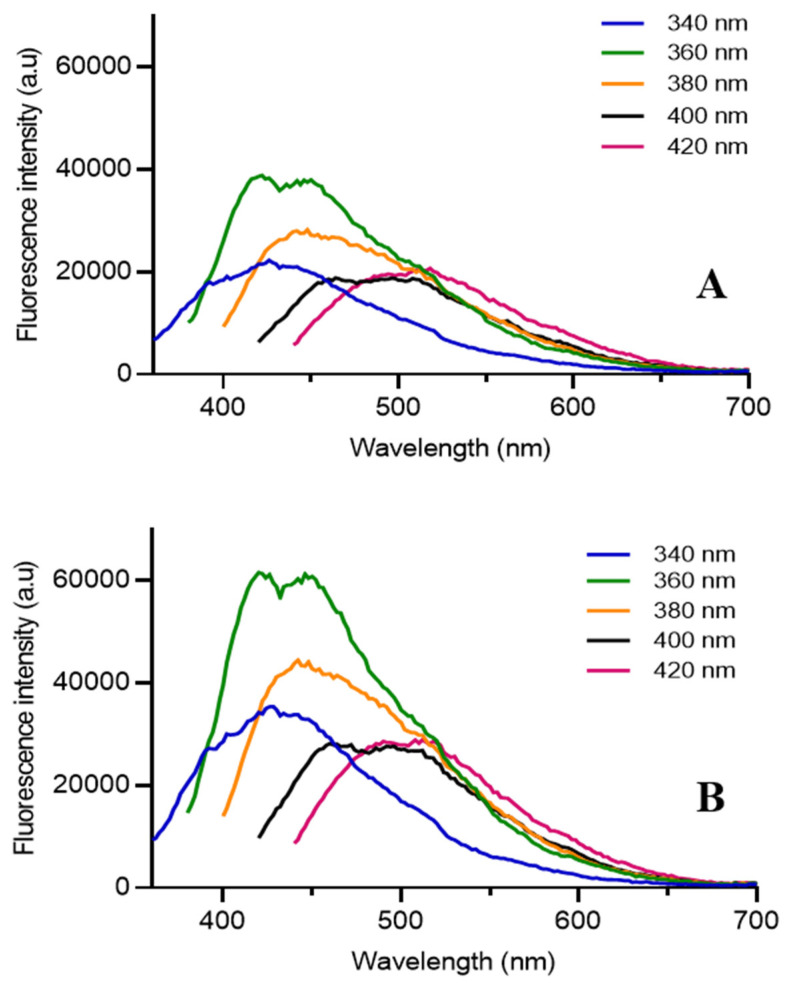
(**A**) Fluorescent emission spectra of CQD-A; (**B**) fluorescent emission spectra of CQD-B.

The emission spectrum (Figure 4) at different excitation wavelengths was recorded to analyze the photoluminescence property of the samples (185 µg/mL). The fluorescence intensity of CQD-B was about 50% higher than CQD-A, and both spectra had similar emission trends with maximum intensity at 360 nm excitation wavelength. Any further increase in the excitation wavelength resulted in the reduction of emission intensity. The emission spectrums also exhibited a redshift and displayed an excitation tunable emission. The excitation dependent emission property of the fabricated CQDs is in agreement with several previous studies reported in the literature [24,38,39]. The fluorescence images of C2C12 cell incubated with CQD-A and CQD-B were brighter and promising with FITC filter which represents the excitation wavelength of 475 to 490 nm. However, the intensity of fluorescence images with TRITC filter was low as its excitation wavelength was 546 to 565 nm. This was in agreement with the emission spectra which exhibited strong emission in green region and weak intensity in red region.

### 3.2. Internalization of CQDs by C2C12 Cells

In bio-medical research, staining is a reliable tool to visualize and enhance features inside cells, tissues, and animal models [40]. Several colored and/or fluorescent stains and dyes like, hematoxylin-eosin, DAPI, fluorescein isothiocyanate, and rhodamine are widely used to achieve this. However, one of the main disadvantages of many of the inherently fluorescent particles is their sheer size, and hence the inability to cross live cell membranes. Most fluorescent dyes are also toxic to live cells, and this necessitates fixing the cells prior to staining. Since the size of synthesized CQDs is less than 10 nm and that they are derived from natural sources with a green route of synthesis, their capacity to cross live cell membranes was tested.

C2C12, the mouse muscle myoblast cell lines, were selected as they are widely used to study muscle formation in vitro. Thus, imaging the myoblasts and myotubes are crucial in this specific research scenario. This can be further applied to other 2D or 3D cell culture model systems, which have the ability to differentiate to several different cell/tissue types across biological systems.

**Figure 5:**
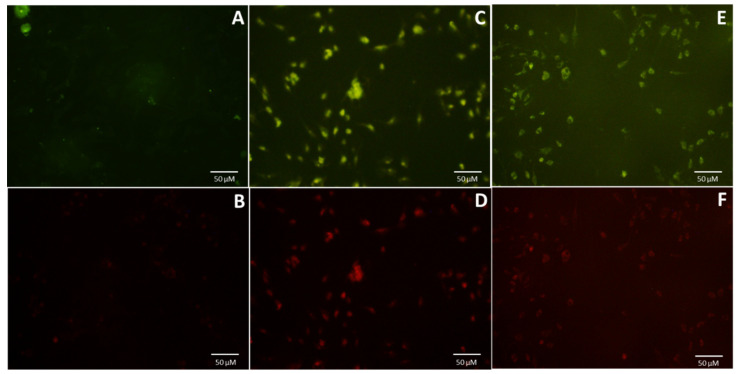
Fluorescence images of C2C12 cells after 24 h of incubation with CQDs. (**A**,**B**) Cells incubated with water as control; (**C**,**D**) cells incubated CQD-A; (**E**,**F**) cells incubated with CQD-B; (**A**,**C**,**E**) are images from FITC (green) channel; (**B**,**D**,**F**) are images from TRITC (red) channel.

C2C12 cells were incubated with the prepared carbon quantum dots, CQD-A and CQD-B. The cells were grown on coverslips pretreated with poly-L-lysine. The CQDs with the concentration of 185 ug/mL were additionally filtered through 0.2 µm filters (to prevent bacterial growth) and added to the media containing cells. Since the CQDs were dissolved in water, the corresponding volume of water was added to the control cells. As the doubling time of C2C12 is 15 h [41], the initial incubations were set at 24 h, which is estimated to be ample time for testing the internalization. After 24 h, the wells containing coverslips were fixed using formalin and after washing with PBS, the coverslips were inverted and mounted onto the slides. The fluorescence imaging was carried out using filters for fluorescein isothiocyanate (FITC) for green and tetramethyl rhodamine (TRITC) for red, depicting the excitation filter wavelengths of 475–490 nm and 545–565 nm respectively. The results (Figure 5) showed a clear internalization of CQDs by C2C12 cells as compared to control cells, both in green and red filters. Since CQD-A yielded better fluorescent intensity when compared to CQD-B (Figure 5A,C,E), CQD-A was chosen for further experiments. Significant cell death was not observed during this experiment making the CQDs important candidates for bio-imaging.

**Figure 6:**
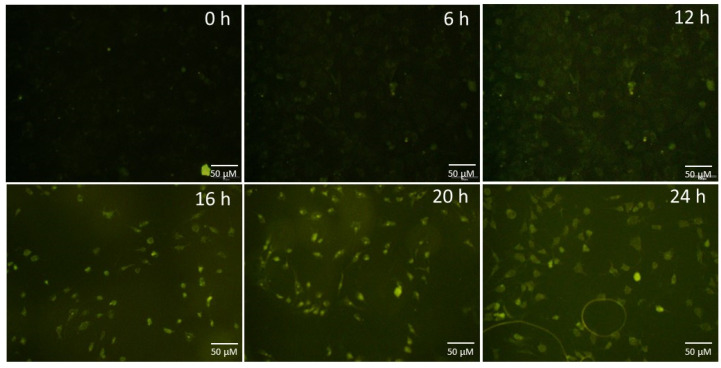
The time course of the internalization of CQD-A by C2C12 cells tracked for 0–24 h. The images were recorded using FITC channel.

Having observed the successful internalization of CQDs by C2C12 cells, the time period taken for this was followed by incubating C2C12 cells with CQD-A for different time intervals (0, 6, 12, 16, 20, and 24 h). The data (Figure 6) showed that as the end of 16 h, the cells exhibited a remarkable change in fluorescence intensity when compared to the control group. This also gives us an estimate of incubation time required for quantum dots with live cells for the purpose of bio-imaging. From the data, 16 h or overnight of incubation is deemed sufficient, thus making them perfect candidates for applications in bio-imaging.

### 3.3. Cytotoxicity

**Figure 7:**
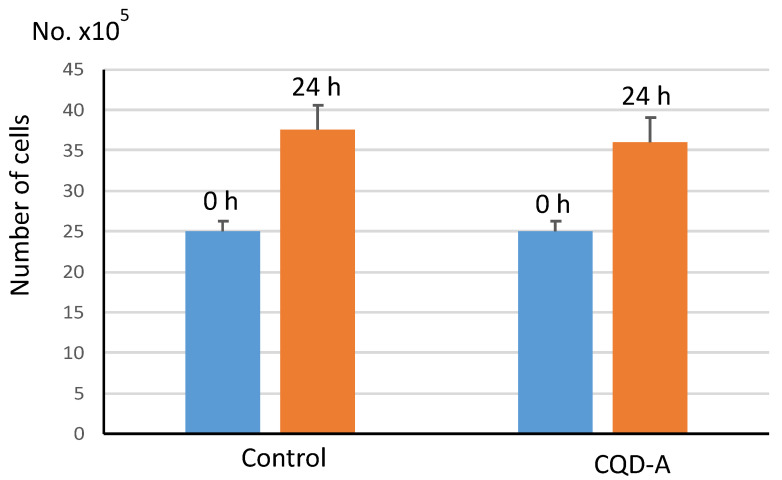
Comparison of cell viability after incubating C2C12 cells with CQD-A for 24 h. The blue bar represents the number of cells counted at 0 h and the orange bar represents the cells counted after 24 h of incubation with either CQD-A or water as a control.

Since low cytotoxicity and resultant minimal cell death is one of the crucial advantages when imaging cells using CQDs, we further estimated cell death after incubating C2C12 cells with CQD-A. For this, 15,000 cells were seeded to each of the cell culture wells. After 24 h of incubation, CQD-A was added. The cells were counted before and 24 h after the addition of CQDs. The results (Figure 7) showed that the CQD-A exhibited very low cytotoxicity. More than 96% of the cells were alive after incubation of 24 h. This is in agreement with several studies published earlier using CQDs derived from natural sources [8,9,42]. Considering that the doubling time of C2C12 cells is 15 h [41], an incubation period of 24 h with CQDS was found to be ample for the purpose. Furthermore, the minimal extent of cell death through cyto-toxicological effect, as exhibited by C2C12 cells in the presence of CQD-A, is negligible. This further confirms that the synthesized CQDs can be used safely for imaging of cell culture systems, and for other biological applications.

### 3.4. Uptake of CQDs by Muscle Myotube and Imaging

Two-dimensional and three-dimensional cell culture model systems, apart from helping to elucidate basic biochemical mechanisms, have tremendous potential in in vitro disease models, tissue engineering, regenerative medicine, cell therapy, as well as for pharmaceutical applications, especially, in drug discovery and development [43]. A simple, low cost, effective, and rapid staining method, therefore, is crucial in different stages of these studies. Evidently, C2C12 cells are one such model system used to study muscle myotube formation from myoblasts. Hence, staining different stages of myotube formation becomes vital in these studies and, if successful, can be studied and applied to other in vitro cell culture model systems and can also be further extended to animal models.

**Figure 8:**
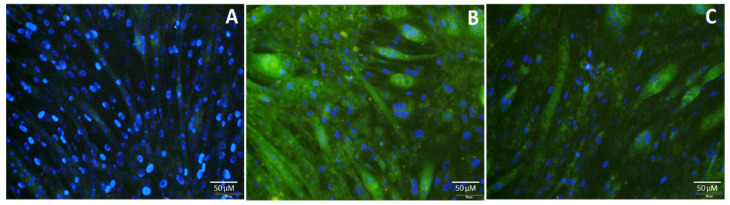
Fluorescence image of C2C12 myotubes incubated with CQDs. (**A**) Control cells; (**B**) myotubes incubated for 24 h with CQD-A; (**C**) myotubes incubated for 24 h with CQD-B. The images are from FITC (green) and DAPI (blue) channels overlaid.

In order to test the ability of CQDS to stain myotubes, C2C12 cell myoblasts were cultured in differentiation media to form myotubes. Several myoblasts fuse to form mature myotubes, thus giving the appearance of multinucleated tubes. After the formation of myotubes in differentiation media, CQD-A was added to the media containing myotubes and incubated for 24 h. The cells were counterstained with nuclear specific stain DAPI to visualize the multinucleated structure. The Figure 8 shows that the CQDs were able to cross the membrane of myotubes and depict a clear image of myotubes without any additional staining methods to visualize them.

These experiments reveal the fluorescence characteristics of the CQDs and that they are easily internalized, by both myoblasts and myotubes, illustrating their use in biological imaging. They are also made from inexpensive sources, have very low cytotoxicity to live cells and human beings, and require incubation time of only 24 h, making them cheaper and safer alternatives to the commercial fluorescent dyes used in biological imaging.

## 4. Conclusions

Carbon quantum dots were fabricated from bread using a thermal process, and in the absence of any undesirable chemicals, for the first time, and the resultant products were purified and characterized. These CQDs exhibited excellent fluorescence and other favorable physical and chemical properties. The intrinsic fluorescent property of the CQDs was successfully made use for imaging of C2C12 cells. The cytotoxicity of these biocompatible CQDs was found to be comparatively low, and hence are excellent candidates for fluorescent bio-imaging sensors. This attribute was further extended and successfully tested to image differentiated C2C12 myotubes. Given that the fabrication process employed in the present work does not resort to conventional wet-chemistry procedures, this research will open a new perspective in the subject area that warrants further investigations for optimizing and scaling up the production of CQDs from bread. The preliminary results from bio-imaging experiments also showed the potential applications of the CQDs, which should be extended to encompass various cell/tissue culture systems and small animal models.

## Supplementary Materials

The following are available online at https://www.mdpi.com/2079-4991/10/8/1575/s1, Figure S1: Contrast enhanced images of TEM. These images were used to count the CQDs for size distribution histogram.
