# Supplementary material for: Bioimaging of C2C12 Muscle Myoblasts Using Fluorescent Carbon Quantum Dots Synthesized from Bread
Source: Nanomaterials (Basel). 2020 Aug 11;10(8):1575. doi: 10.3390/nano10081575 (PMC7466409; doi:10.3390/nano10081575)
Supplement: Supplementary file 1 [file nanomaterials-10-01575-s001.pdf]

nanomaterials

Supplementary Materials: Bioimaging of C2C12 Muscle Myoblasts Using Fluorescent Carbon Quantum Dots Synthesized from Bread

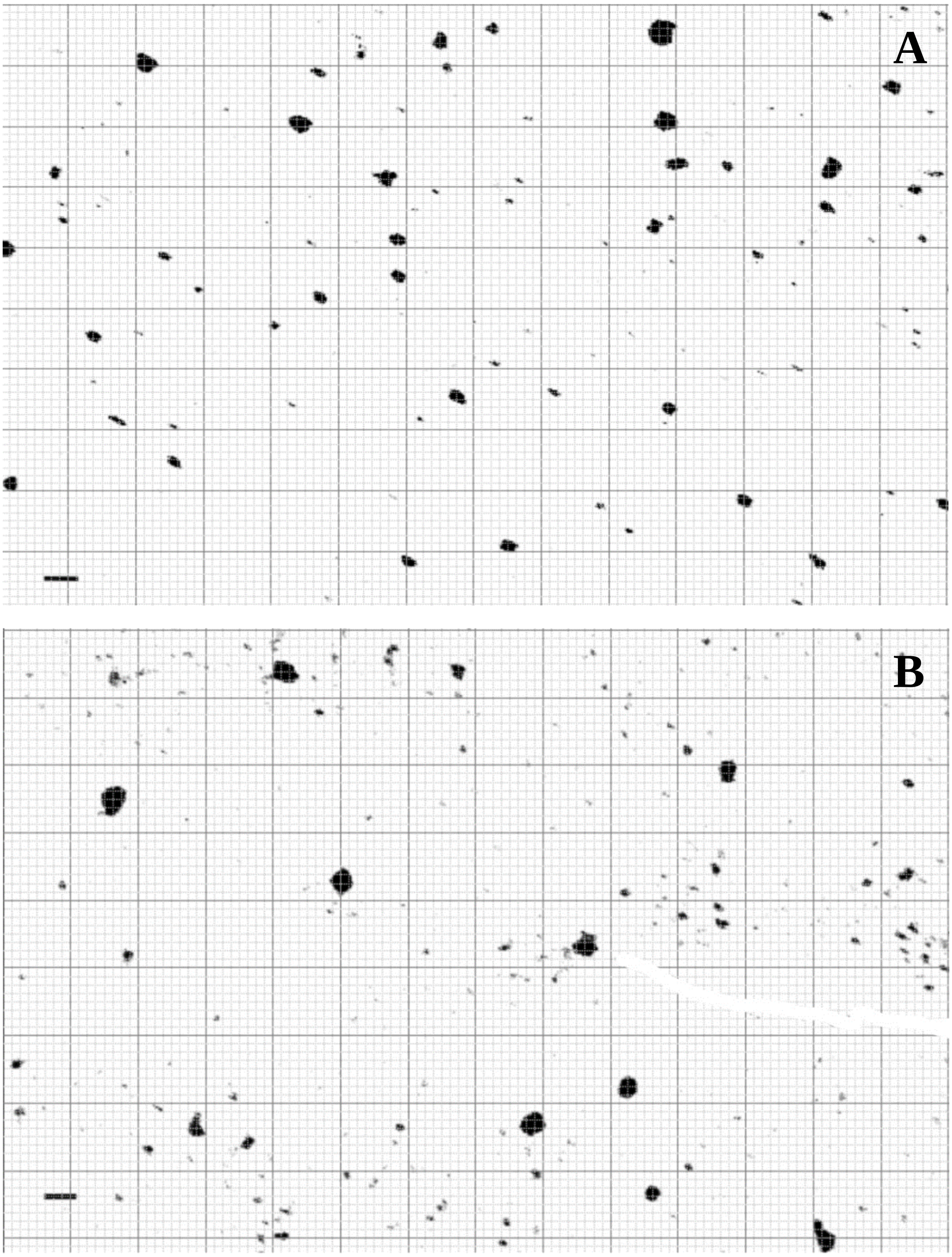

Figure S1. The contrast enhanced TEM images of CQD-A (**A**) and CQD-B (**B**).
